# The Impact of Home Medication Management Practices on Medication Adherence

**DOI:** 10.3390/bs14090804

**Published:** 2024-09-11

**Authors:** Lisa Gualtieri, Meredith Steinfeldt, Eden Shaveet, Brandon Estime, Meera Singhal

**Affiliations:** 1 Tufts University School of Medicine, Boston, MA 02111, USA; 2 Nicklaus Children’s Health System, Miami, FL 33155, USA; meredith.steinfeldt@nicklaushealth.org; 3 Cornell University, New York, NY 10044, USA; ems486@cornell.edu; 4 Children’s National Hospital, Washington, DC 20010, USA; bestime@childrensnational.org; 5 Lewis Katz School of Medicine, Temple University, Philadelphia, PA 19140, USA; meera.singhal@temple.edu

**Keywords:** medication adherence, home medication management, survey, digital health, user-centered design

## Abstract

Medication adherence is a vexing challenge, with over 50% of US adults not adhering to their prescribed medication regimen. Most medications are self-administered by patients at home, requiring them to independently develop and manage their own medication routines. By understanding these home-based practices, such as where patients store their medications and how different storage locations impact adherence, we can develop targeted interventions to improve adherence rates. Our goal was to identify and categorize self-reported home medication management practices and determine which practices are associated with self-reported medication adherence. From the 1673 total survey respondents we learned that the most common places people store their medications at home are nightstand drawers (28%), on top of nightstands (27%), kitchen cabinets (22%), and bathroom medicine cabinets (20%). Kitchen cabinets and bathroom vanities were significantly associated with increased odds of having ever forgotten to take a medication. On the other hand, desks, dining room tables, and the inside of nightstand drawers were associated with the greatest statistically significant decrease in odds of having ever forgotten to take a medication. Almost all (96%) respondents were receptive to receiving guidance from healthcare providers on how to store medications. Patients are largely responsible for creating their own home medication management practices, including deciding where to store their medication at home. Understanding which home storage locations are associated with medication adherence can lead to innovative approaches to improve adherence, including personalized guidance to patients from their healthcare providers for patients.

## 1. Introduction

### 1.1. Definition and Importance

Medication adherence, defined as the extent to which patients take medication as prescribed, was highlighted as “a problem of striking magnitude” by the World Health Organization (WHO) [1]. Studies indicate that 50% or fewer US adults are adherent to their medication, which has contributed to deaths and hospital admissions [2,3,4]. The problem of medication nonadherence is growing across all ages, racial/ethnic groups, and sexes [5]. With the prevalence of nonadherence and the severity of the health consequences, the WHO states that enhancing the efficacy of medication adherence interventions could have a more significant impact on population health than advancements in specific medical treatments [6].

### 1.2. Medication Adherence

More medications are self-administered at home than in hospitals and clinics combined [7]. Consequently, understanding home medication management is critical for improving medication adherence. Numerous barriers to medication adherence have been identified, including medication cost, limited access to pharmacies, and poor health literacy skills [8,9,10,11,12]. Adherence is also influenced by factors that evolve as a patient progresses through different phases of their medication regimen [13]. The Ascertaining Barriers to Compliance (ABC) taxonomy identifies the stages of adherence as initiation, which is the consumption of the first dose of a prescribed medication; implementation, which refers to adherence to the prescribed dosing regimen from initiation until the final dose of the medication; and discontinuation, which occurs when a patient stops taking the medication for any reason [14]. The taxonomy further distinguishes between intentional and unintentional nonadherence to illustrate the complex nature of adherence [15]. One of the most common unintentional reasons for nonadherence is forgetfulness [16].

Throughout the stages of the medication lifecycle, healthcare providers have multiple opportunities to guide patients in establishing or improving medication management practices. Given the in-depth knowledge providers have of their patient’s health and medication histories, coupled with the trust patients place in them, they are arguably the best sources for this guidance [17,18]. Interventions to improve adherence also include pharmacist counseling and home visits to provide tailored guidance on medication regimens [19,20,21]. However, most patients have limited opportunities to receive guidance due to constraints on provider time and the costs associated with these interventions. Guidance can also come from family and friends, and studies have shown the value of leveraging social contacts to engage patients in effective medication management [22]. For family and friends to be helpful, they need prior experience with adherence, comfort with discussing medication-related issues, and availability for ongoing communication.

Most patients must independently devise and implement their own home medication management practices, including decisions on where to store medications, how to establish medication-taking routines, and which containers, devices, or apps to use for adherence support. At the initiation phase, they may have little knowledge to build on.

Categorizing medication storage locations and determining which are associated with adherence are an understudied area [23]. Existing research and patient guidelines on storage primarily focus on safety, including how to store medications securely in homes with children or pets [13], avoiding environmental factors like heat, humidity, and light that can compromise medication stability [24]; preventing medication errors [14]; and reducing the risk of medication misuse due to poor medication management [15].

We describe our efforts to identify and categorize home medication management practices as a foundational step toward developing interventions aimed at improving medication adherence in patients’ homes [25]. Other than prior studies on medication safety, this study is unique in identifying home storage locations and reporting which of these locations are associated with better medication adherence.

## 2. Methods

### 2.1. Study Sample

We designed a survey to learn about home medication management. Data were collected via participant self-reports through an online survey that was administered in English using Google Forms, an online survey platform that uses data encryption and advanced malware protection to protect user responses [26]. After the survey was designed and piloted, all protocols were submitted to and approved by the Tufts University Health Sciences Institutional Review Board (IRB) under STUDY00002224.

The survey was open between 18 November and 14 December 2021, and participants were recruited using social media platforms (Twitter, LinkedIn, Facebook, and Instagram) and through Tufts University’s Osher Lifelong Learning Institute mailing list. Eligible participants were 18 years of age or older. Informed consent was obtained from all subjects. Anyone who completed the survey was eligible to win one of ten USD $25 Amazon gift cards by random draw.

### 2.2. Procedures

A total of 1966 survey responses were collected. Of those, 1673 (85%) responses were deemed valid after screening for exclusion criteria (n = 293, 15%). Responses were dropped if they had any of the following qualities: consecutive sets of identical responses reported at the same time (n = 132); suspicious responses including identical open-text responses from the same day (n = 83); non-English responses (n = 59); or responses reported using an abnormal email address containing long strings of numbers, which were suspected to be fraudulently generated (n = 19).

### 2.3. Measures

The items included in our survey were designed to elicit respondents’ experiences with managing their medication at home. The questions were either multiple choice, on a 5-point Likert scale, or free-text open-ended to collect participant experiences related to medication storage locations, self-reported medication adherence, and perceived importance of adherence. Data pertaining to age, race, ethnicity, sex, and highest level of education were also collected.

### 2.4. Medication Storage Location

One of the survey questions, “Where in your home do you store your prescriptions that you take on a regular basis?”, was designed to elicit where respondents stored the medications taken regularly. Because an individual might use multiple locations, respondents could select one or more locations; the options were as follows: “Kitchen table”, “Kitchen cabinet”, “Kitchen counter”, “Kitchen drawer”, “In the refrigerator”, “On the bathroom vanity”, “In the vanity drawer or cabinet”, “Bathroom medicine cabinet”, “On top of the bedroom nightstand”, “In the nightstand drawer”, “Desk”, “Dining room table”, “Backpack, purse, or bag”, and “Closet”. An open text field was included for locations not in the list. Open-text responses were categorized by existing values via consensus coding to promote inter-rater reliability.

A separate question asked where respondents stored medications that they did not regularly take: “Where in your home do you store medications other than the ones that you take on a regular basis (such as over-the-counter medications, an extra supply of your medications, vitamins, etc.)?” Respondents could select from the same options provided above.

### 2.5. Analysis

The analysis of survey results assessed relative frequencies for all sample characteristics and variables listed in Measures. Bivariate analyses were performed for the home medication storage locations used by survey respondents, the perceived importance of medication adherence, and the variables indicating adherence to medications taken regularly. Bivariate analyses included chi^2^ tests of homogeneity of proportions and bivariate logistic regression models.

## 3. Results

### 3.1. Sample Demographic Characteristics

Of the 1673 total respondents, the ages ranged from 18 to over 90 years old with most (65%) being between the ages of 20 and 39 years. For race/ethnicity, a majority identified as White (62%). For sex, over half (55%) identified as female. For the highest degree obtained, a majority (87%) had at least some college coursework experience with 45% having obtained a bachelor’s, master’s, or professional degree. The distribution of the sample is depicted in Table 1.

### 3.2. Receptivity to Storage Guidance

Over 1600 respondents (N = 1606, 96%) reported being receptive to guidance on medication storage, with more than half (54%) saying they would be open to receiving guidance if it was from a physician. More respondents (60%) said they would be receptive to guidance from a pharmacist. Fewer said they would be open to receiving guidance from a mobile application or website (18%) or would be receptive to printed material, such as brochures (13%). Nearly two-thirds (65%) of those who reported being receptive to guidance from a physician said that they had not recently forgotten to take a medication (*p* = 0.026), while 54% of those who were open to guidance from a mobile application or website self-reported that they had not recently forgotten to take a medication (*p* = 0.002).

### 3.3. Storage Location

The most common home storage location for primary medications was in nightstand drawers (N = 475, 28%), followed closely by atop bedroom nightstands (N = 456, 27%). Other common storage locations for primary medications were kitchen cabinets (N = 372, 22%) and bathroom medicine cabinets (N = 330, 20%). Among these commonly reported storage locations, atop bedroom nightstands (*p* = 0.005), nightstand drawers (*p* < 0.001), and kitchen cabinets (*p*= 0.001), significantly fewer respondents reported recently forgetting to take their medication. Other storage locations for primary medications that had significantly fewer respondents recently forgetting to take their medication included the kitchen counter (*p* = 0.013), desk (*p* = 0.040), dining room table (*p* = 0.042), and closet (*p* = 0.004). Table 2 shows the primary medication storage locations for our sample.

The most common reasons respondents chose their medication storage locations were related to medication safety, visibility, and proximity. The majority of those who chose a storage location due to safety (*p* < 0.001), visibility (*p* = 0.003), proximity to items used daily (*p* = 0.001), proximity to food or drink (*p* = 0.017), and household conditions (*p* = 0.017) reported that they had not recently forgotten to take their medication. Those who chose safety were also less likely to report ever forgetting to take their medication (*p* < 0.001).

### 3.4. Bivariate Analyses

#### Medication Storage Location and Ever or Recently Forgetting to Take a Medication

The home medication storage locations that were significantly associated with decreased odds of having ever forgotten to take a medication included kitchen tables (OR: 0.67, 95% CI: 0.50–0.88), kitchen drawers (OR: 0.72, 95% CI: 0.56–0.93), in the refrigerator (OR: 0.71, 95% CI: 0.55–0.91), atop the bedroom nightstand (OR: 0.58, 95%CI: 0.46–0.72), in the nightstand drawer (OR: 0.52, 95% CI: 0.42–0.65), desks (OR: 0.48, 95% CI: 0.37–0.63), dining room tables (OR: 0.51, 95% CI: 0.37–0.71), and a backpack, purse, or bag (OR: 0.70, 95% CI: 0.53–0.92). The two medication storage locations that were significantly associated with increased odds of having ever forgotten to take a medication were kitchen cabinets (OR: 1.39, 95% CI: 1.09–1.76) and bathroom vanities (OR: 2.15, 95% CI: 1.53–3.02).

Five home medication storage locations were significantly associated with having decreased odds of forgetting to take a medication within the two weeks prior to the survey: atop bedroom nightstand (OR: 0.73, 95% CI: 0.58–0.91), in the nightstand drawer (OR: 0.60, 95% CI: 0.48–0.75), desk (OR: 0.75, 95% CI: 0.56–0.99), on the dining room table (OR: 0.70, 95% CI: 0.49–0.99), and in the closet (OR: 0.46, 95% CI: 0.27–0.79). Two locations were significantly associated with increased odds of forgetting to take a medication in the two weeks prior to the survey: kitchen cabinets (OR: 1.48, 95% CI: 1.17–1.87) and kitchen counters (OR: 1.38, 95% CI: 1.17–1.78) This analysis is shown in Table 3.

### 3.5. Additional Findings

Some additional analysis of survey results found that nearly two-thirds (63%) of those who lived alone reported that they had ever forgotten to take their medication, compared to 64% of people living with 1–2 people, 55% of those living with 3–4 people, and 51% of those living with 5 or more people; *p* = 0.002. Over a third of the sample (N = 599, 36%) reported that they were responsible for helping one or more people they lived with manage their medication, while 15% of the sample said they helped one or more people they did not live with. Fewer respondents (N = 137, 8%) reported helping individuals that they did and did not live with, though this group had a significantly low rate of ever forgetting to take their medication (43%, *p* < 0.001).

Nearly half (48%) of the sample reported being reminded to take their medication by a spouse or a partner, the highest frequency among the sample. Over two-thirds of these respondents (68%) said they had not recently forgotten to take a medication. Respondents who reported that no one reminded them to take their medication had the lowest rate of historical medication adherence (only 87% said that they had forgotten to take a medication at least once in their life). These findings suggest that if one lives with others, living with a spouse or partner might be the most significant relationship for supporting adherent behavior.

## 4. Discussion

### 4.1. Receptivity to Storage Guidance

Nearly all (96%) of the respondents were receptive to receiving guidance on medication storage, and more than half (54%) of the sample reported that they would be open to receiving guidance from a physician. Two-thirds (65%) of these respondents reported that they had not recently forgotten to take a medication, indicating that their receptivity was not to reduce nonadherence but rather to improve their medication management practices. Although respondents desired guidance, changes in the healthcare system, such as shorter doctor’s visits, may limit the opportunities for patients to receive counseling on medication adherence.

### 4.2. Storage Location

Our primary goals in planning this study were to learn which locations in the home patients use for medication storage and if any of those locations were associated with adherence. Nightstands were the most common location for respondents to store their medications, with respondents using nightstand drawers (28%) and atop nightstands (27%) more than other locations. Among those commonly used locations, both atop bedroom nightstands and within nightstand drawers were significantly associated with recent medication adherence.

Those who chose a storage location due to safety (43% of the sample) reported the highest rate of medication adherence (for both ever forgetting and recently forgetting). Visibility was also associated with adherence, with 67% of those who said they chose their medication storage location for visibility purposes reporting that they had not recently forgotten to take a medication.

These findings can inform the guidance provided to patients on where to store their medication and can also inform the design of devices tailored for specific storage locations. Additional characteristics of a location, such as whether a storage location is in a personal or shared space and its visibility, should be factored into counseling on home medication management.

## 5. Future Directions

### 5.1. Guidance from Physicians and Pharmacists

With most respondents (96%) expressing interest in receiving guidance, one future direction is to develop interventions to provide patients with personalized guidance on medication storage. Since half of respondents (54%) indicated they would be receptive to guidance from their physician, home medication storage discussions could occur during office visits. This would require physicians to allocate time for a discussion and ask key questions to personalize guidance to both a living situation and any past adherence history. Interventions could address patients’ needs under three sets of circumstances: how to manage their first prescription, how to manage adding a prescription that increases the complexity of a medication regimen, and a review of current adherence practices.

An even larger number (60%) of respondents were interested in receiving guidance from a pharmacist. The combination of a prescription history, including frequency of prescription refills, in combination with key questions about home medication management, can identify patients who are nonadherent. Interventions could use these indicators to prompt pharmacists to provide guidance about home medication storage. However, guidance may require a different format for the almost 25% of the US population who have their medications shipped directly to their home, thereby removing opportunities for face-to-face interaction between patients and pharmacists [27]. Other changes that interventions need to accommodate include fewer prescriptions dropped off because of electronic prescribing and longer waits to talk to pharmacists who are administering vaccines.

### 5.2. Better Home and Device Design for Supporting Adherence

With nightstands being the most common place for respondents to store their medications and being the most significantly associated with recent medication adherence, innovative designs could support medication adherence. There are currently nightstands on the market with a spectrum of features designed to support charging devices and other technology-related activities. Novel nightstand designs could accommodate prescription bottles and pill containers to increase fit and visibility. Other settings in patients’ homes could be designed to support medication adherence. One example is to relocate built-in medicine cabinets to a cooler and drier environment; another is to design a kitchen cabinet specifically for medication much like spice cabinets, which are designed to ease locating and removing containers of spice.

Our findings on where patients store their medications could lead to the design of location-specific containers or devices in the same way that lighting differs depending on location. A desktop lamp, a living room lamp, and a kitchen light all work for a specific setting. Another important consideration is that many digital devices use auditory or visual cues to provide time-based reminders to patients to take their medication. However, these reminders are only effective if a patient is in a position to see or hear them. To improve adherence, these alerts could be redesigned to align with home usage patterns, ensuring that reminders are noticed. Time-based notifications may not even be an effective approach for adherence reminders, especially when most medications do not need to be taken at a specific time and most patients have fluctuations in their schedules. A promising future direction is to apply principles similar to car seatbelts alerts, which emit an auditory notification if the seatbelt is unlatched when the ignition is turned on. This concept could be adapted for medication adherence by pairing sensors on prescription bottles or pill containers with sensors tied to everyday routines, such as making coffee or brushing teeth, to provide reminders precisely when they are needed [28].

## 6. Limitations

Using social media as our primary survey dissemination method restricted our respondent pool to those active on the platforms we utilized. Additionally, while the survey methodology was useful for gathering self-reported data, it did not allow for a deeper exploration of the underlying choices and behaviors. Despite these limitations, we successfully identified significant bivariate relationships between several rationales respondents cited for their choice of medication storage locations and their self-reported adherence.

## 7. Conclusions

This survey uncovered where people store their medications and identified which storage locations are associated with medication adherence. Other results center on the factors influencing patients’ choice of medication storage locations and their receptivity to receiving guidance. These findings have inspired the development of interventions for healthcare professionals to provide adherence guidance, as well as innovative approaches to the design of homes, furnishings, and devices tailored to home medication management. The survey has further led to a follow-up study using in-depth interviews to explore the complex decisions patients make in managing their medications at home [29].

## Figures and Tables

**Table 1:** Characteristics of sample by self-reported adherence (n = 1673).

Characteristic	Overall N (%)	Ever Forgot to Take a Medication (Row %)				Recently Forgot to Take a Medication ^a^ (Row %)			
		Yes	No	Chi^2^ *	*p*	Yes	No	Chi^2^ *	*p*
**Overall**	1673 (100)	60	40			38	62		
**Age by decade**									
<19	9 (<1)	67	33	81.44	<0.001	67	33	17.39	0.026
20–29	443 (26)	55	45			42	58		
30–39	641 (38)	54	46			38	62		
40–49	244 (15)	59	41			39	61		
50–59	127 (8)	75	25			35	65		
60–69	82 (5)	85	15			37	63		
70–79	84 (5)	86	14			23	77		
80–89 ^b^	38 (2)	82	18			29	71		
90 ^b^	5 (<1)	80	20			20	80		
**Race/ethnicity ^c^**									
White	1040 (62)	61	39	0.38	0.536	35	65	12.86	<0.001
American Indian or Alaskan Native	254 (15)	47	53	20.95	<0.001	67	33	2.43	0.119
Black or African American	168 (10)	68	32	5.32	0.021	51	49	14.16	<0.001
Asian	138 (8)	68	32	3.94	0.047	54	46	15.94	<0.001
Hispanic or Latino	136 (8)	60	40	<0.01	0.980	46	54	4.53	0.033
Native Hawaiian or Other Pacific Islander	68 (4)	81	19	12.66	<0.001	66	34	24.20	<0.001
**Sex ^d^**									
Female	910 (55)	64	36	19.92	<0.001	38	62	4.91	0.086
Male	765 (45)	55	45			37	63		
**Highest education completed**									
<High school diploma or equivalency	47 (3)	75	26	43.77	<0.001	62	38	21.63	0.001
High school diploma or equivalency	174 (10)	55	45			43	57		
Some college	444 (27)	50	50			36	64		
Associate's degree	182 (11)	56	44			45	55		
Bachelor's degree	390 (23)	65	35			34	65		
Master’s or professional degree ^b^	365 (22)	68	32			36	64		
Doctoral degree	71 (4)	73	27			34	66		
**Receptivity to storage guidance ^c^**									
Receptive to guidance from at least one ^e^	1606 (96)	60	40	0.87	0.350	38	62	2.67	0.103
From physician	909 (54)	57	43	6.35	0.012	35	65	4.93	0.026
From pharmacist	996 (60)	62	38	4.78	0.029	37	63	1.24	0.265
From mobile application or website	304 (18)	75	25	32.51	<0.001	46	54	9.83	0.002
From printed material (brochures)	221 (13)	76	24	28.16	<0.001	39	61	0.13	0.723
Not receptive to guidance from any listed source ^f^	67 (4)	66	34	0.87	0.350	28	72	2.67	0.103
**Reason for choosing storage location**									
Safety	714 (43)	47	53	84.01	<0.001	32	68	20.25	<0.001
Ease of access	620 (37)	73	27	69.85	<0.001	40	60	1.42	0.233
Visibility	612 (37)	53	47	21.17	<0.001	33	67	8.93	0.003
Proximity to items used every day	560 (33)	66	34	11.42	0.001	43	57	10.35	0.001
Proximity to food or drink	391 (23)	60	40	2.23	0.135	43	57	5.71	0.017
Household conditions	282 (17)	55	45	3.87	0.049	32	68	5.68	0.017
Other	3 (<1)	100	0	1.99	0.159	0	100	1.83	0.176

^a^: “Recently” was defined as forgetting to take a medication in the last two weeks. ^b^: These values were combined due to the small sample size or similarity. ^c^: These were not mutually exclusive; a single respondent may fall into several categories, except for composite values. ^d^: <1% identified with a sex that was unlisted. ^e^: Composite variable or value. ^f^: Derived from absence of value selection. * Pearson chi^2^: The *p*-values denote likelihood of observed chi^2^, assuming homogeneity of the proportions. For the non-mutually exclusive items, chi^2^ is calculated for each selection as a binary value by the binary adherence proxy.

**Table 2:** Home medication storage by ever or recently forgetting to take medication (n = 1673).

Home Medication Storage Locations	Overall N (%)	Ever Forgot to Take a Medication (Row %)				Recently Forgot to Take a Medication ** (Row %)			
		Yes	No	Chi^2^ *	*p*	Yes	No	Chi^2^ *	*p*
Overall	1673 (100)	60	40			38	62		
Kitchen table	223 (13)	52	48	7.98	0.005	42	58	1.63	0.201
Kitchen cabinet	372 (22)	66	34	7.04	0.008	45	55	10.91	0.001
Kitchen counter	294 (18)	62	38	0.28	0.596	44	56	6.18	0.013
Kitchen drawer	298 (18)	54	46	6.39	0.011	42	58	3.05	0.081
In refrigerator	284 (17)	53	47	7.04	0.008	39	61	0.37	0.542
On bathroom vanity	196 (12)	75	25	20.32	<0.001	41	59	1.15	0.284
In vanity drawer or cabinet	245 (15)	61	39	0.05	0.829	38	62	<0.01	0.966
Bathroom medicine cabinet	330 (20)	62	38	0.86	0.355	36	64	0.76	0.385
Atop bedroom nightstand	456 (27)	50	50	24.88	<0.001	32	68	7.71	0.005
In the nightstand drawer	475 (28)	49	51	35.66	<0.001	29	71	19.72	<0.001
Desk	264 (16)	45	55	29.89	<0.001	32	68	4.24	0.040
Dining room table	161 (10)	45	55	16.39	<0.001	30	70	4.15	0.042
Backpack, purse, or bag	239 (14)	53	47	6.50	0.011	35	65	0.86	0.354
Closet	80 (5)	51	49	2.80	0.094	22	78	8.40	0.004
Unlisted location (Alone) ***	17 (1)	94	6	8.25	0.004	35	65	0.05	0.828

* *p*-values denote the likelihood of observed chi^2^, assuming homogeneity of the proportions. For all values, chi^2^ is calculated for each selection as a binary value (only affirmative displayed) by the binary adherence proxy. ** “Recently” is defined as forgetting to take a medication in the last two weeks. *** Inclusive of uncategorizable responses for which a categorizable response was not also provided.

**Table 3:** Bivariate associations between home medication storage location and self-reported medication adherence.

Home Medication Storage Location	Ever Forgot to Take a Medication (Row %)	Recently Forgot to Take a Medication ** (Row %)
	OR (95% CI)	OR (95% CI)
Kitchen table	0.67 (0.50–0.88) *	1.21 (0.91–1.61)
Kitchen cabinet	1.39 (1.09–1.76) *	1.48 (1.17–1.87) *
Kitchen counter	1.07 (0.83–1.39)	1.38 (1.07–1.78) *
Kitchen drawer	0.72 (0.56–0.93) *	1.25 (0.97–1.62)
In refrigerator	0.71 (0.55–0.91) *	1.08 (0.84–1.41)
On bathroom vanity	2.15 (1.53–3.02) *	1.18 (0.87–1.60)
In vanity drawer or cabinet	1.03 (0.78–1.36)	1.01 (0.76–1.33)
Bathroom medicine cabinet	1.12 (0.88–1.44)	0.89 (0.70–1.15)
Atop bedroom nightstand	0.58 (0.46–0.72) *	0.73 (0.58–0.91) *
In nightstand drawer	0.52 (0.42–0.65) *	0.60 (0.48–0.75) *
Desk	0.48 (0.37–0.63) *	0.75 (0.56–0.99) *
Dining room table	0.51 (0.37–0.71) *	0.70 (0.49–0.99) *
Backpack, purse, or bag	0.70 (0.53–0.92) *	0.87 (0.66–1.16)
Closet	0.68 (0.44–1.07)	0.46 (0.27–0.79) *

* *p* < 0.05. ** “Recently” is defined as forgetting to take a medication in the last two weeks.

## Data Availability

The data that support the findings of this study are available from the corresponding author, L.G., upon reasonable request.
